# Supplementary material for: Human neuroepithelial stem cell regional specificity enables spinal cord repair through a relay circuit
Source: Nat Commun. 2018 Aug 24;9:3419. doi: 10.1038/s41467-018-05844-8 (PMC6109094; doi:10.1038/s41467-018-05844-8)
Supplement: Supplementary file 1 — Supplementary Information [file 41467_2018_5844_MOESM1_ESM.pdf]

Supplementary Information

Human Neuroepithelial Stem Cell Regional Specificity Enables Spinal Cord Repair through a Relay Circuit

Dell'Anno et al.

Supplementary Table 1: passive and active properties of SC-NES cell-derived neurons

**Properties**	**SC Neurons (n = 10)**
Membrane capacitance (pF)	49.7 ± 7.2
Membrane Resistance (MΩ)	323 ± 55
Membrane Voltage (mV)	-57.1 ± 1.6
Spike Threshold (mV)	-47.9 ± 1.6
Spike Amplitude (mV)	76.6 ± 3.6
Spike Duration (ms)	1.77 ± 0.09
Sodium Current Density (pA/pF)	-158 ± 21

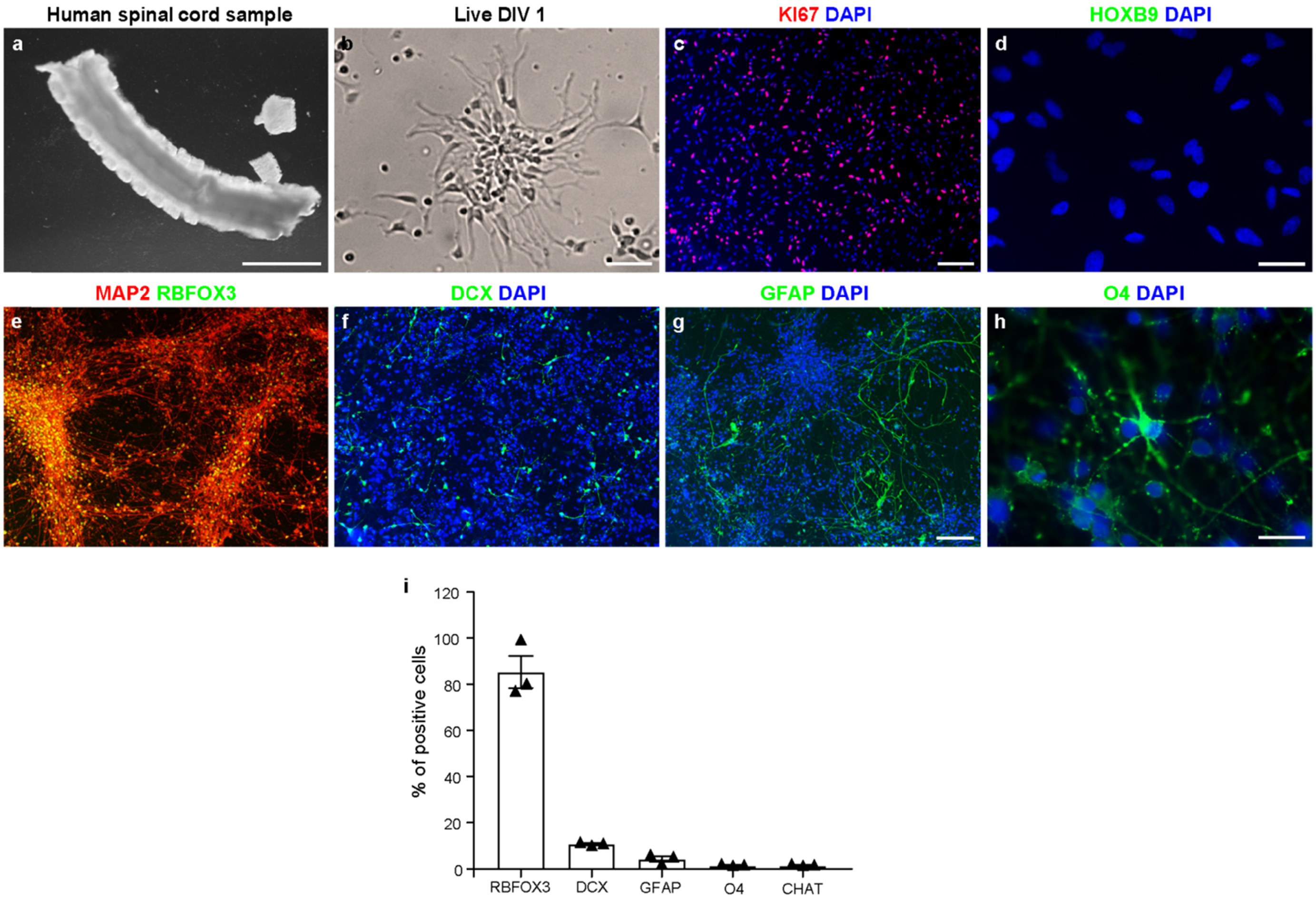

Supplementary Fig. 1. Human SC-NES cells characterization *in vitro*

(a) Bright field contrast image of a human SC segment at 6 PCW. (b) Bright field contrast image of a SC-NES cell rosette after one day from derivation. (c) KI67 staining showing a fraction of cells under proliferation. (d) NCX-NES cells are negative for HOXB9. (e) SC-NES cells generate MAP2- and RBFOX3-positive neurons. (f) After differentiation a small fraction of cells is positive for the early post-mitotic neuronal marker doublecortin (DCX). (g,h) SC-NES cells are tripotent and give rise to GFAP-positive astrocytes and O4-positive oligodendrocytes, too. (i) Quantification of RBFOX3-positive neurons, DCX-positive early post mitotic neurons, GFAP-positive astrocytes, O4 positive-oligodendrocytes and CHAT-positive neurons. Data represent mean ± s.e.m. Scale bars: a, 500 µm; b, 40 µm; c, 100 µm; d, 20 µm; e-g, 100 µm; h, 20 µm.

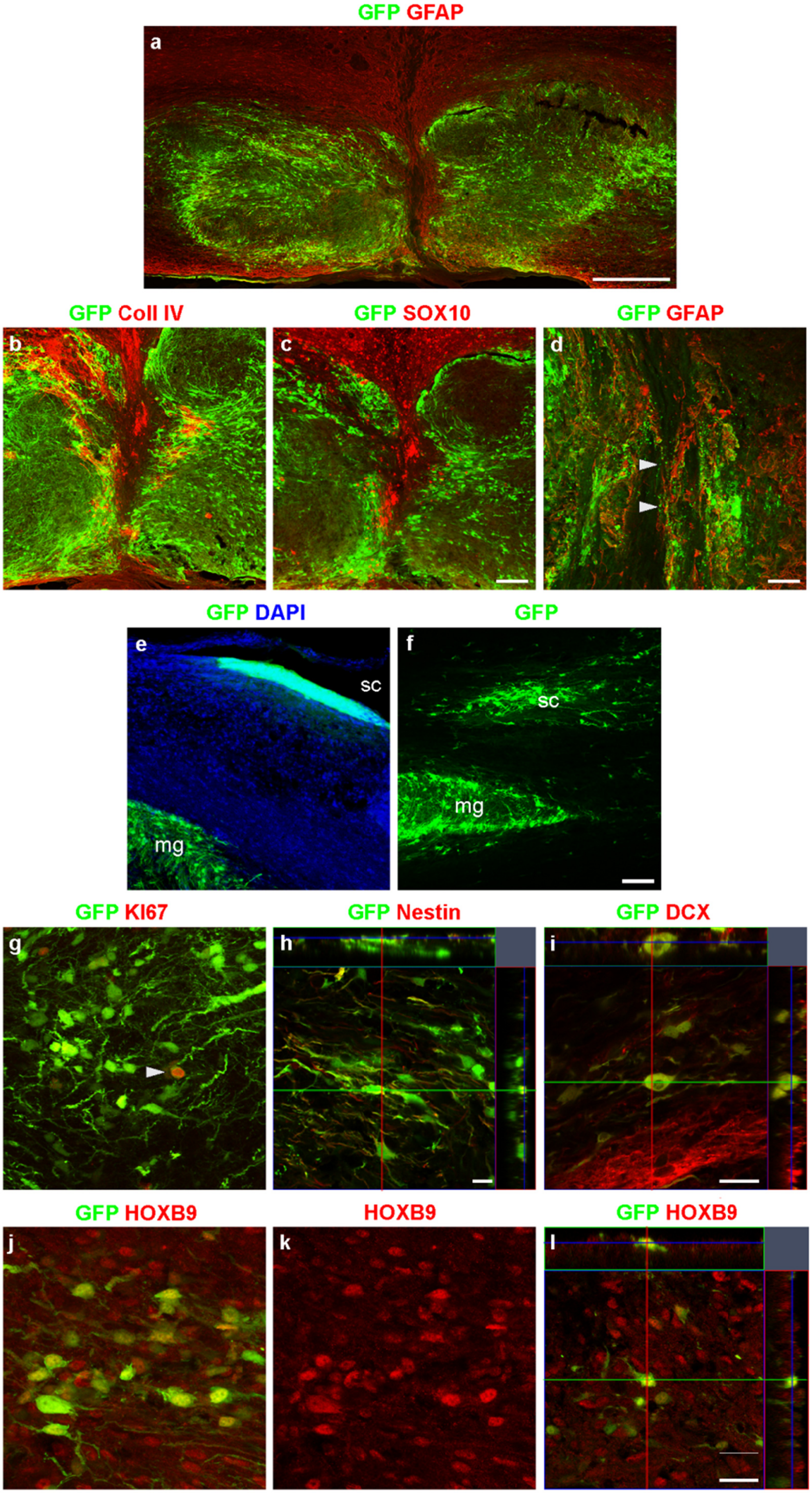
GFP GFAP
a
GFP Coll IV
b
GFP SOX10
c
GFP GFAP
d
GFP DAPI
e
sc
mg
GFP
f
sc
mg
GFP KI67
g
GFP Nestin
h
GFP DCX
i
GFP HOXB9
j
HOXB9
k
GFP HOXB9
l

Supplementary Fig. 2. Human SC-NES graft morphology and cellular characterization

(a) GFP and GFAP staining of SC-NES cells on a SC horizontal section. The staining reveals the presence of a rift in 3 out of 9 recipient animals which segregates the graft into rostral and caudal components. (b,c) The rift appears to be filled with collagen (Coll IV) and SOX10-positive Schwann cells from the host. (d) In 1 out of 3 recipient mice exhibiting a rift we visualize few GFP axons crossing the gap. (e, f) Grafted cells can migrate from the original injection site (main graft; mg) and form satellite clusters (sc) below meninges (e), or in the surrounding parenchyma (f). (g-i) KI67 staining reveals that a fraction of grafted SC-NES cells can still be in a proliferative status and does not acquire a fully differentiated phenotype as attested by the presence of nestin- and doublecortin (DCX)-positive cells. (j-l) Grafted SC-NES cells retain their positional identity *in vivo* as proved by the immunostainings for HOXB9. Scale bars: a, 500 µm; b,c,e,f, 100 µm; d, 50 µm; g-h, 10 µm; i-l, 20 µm.

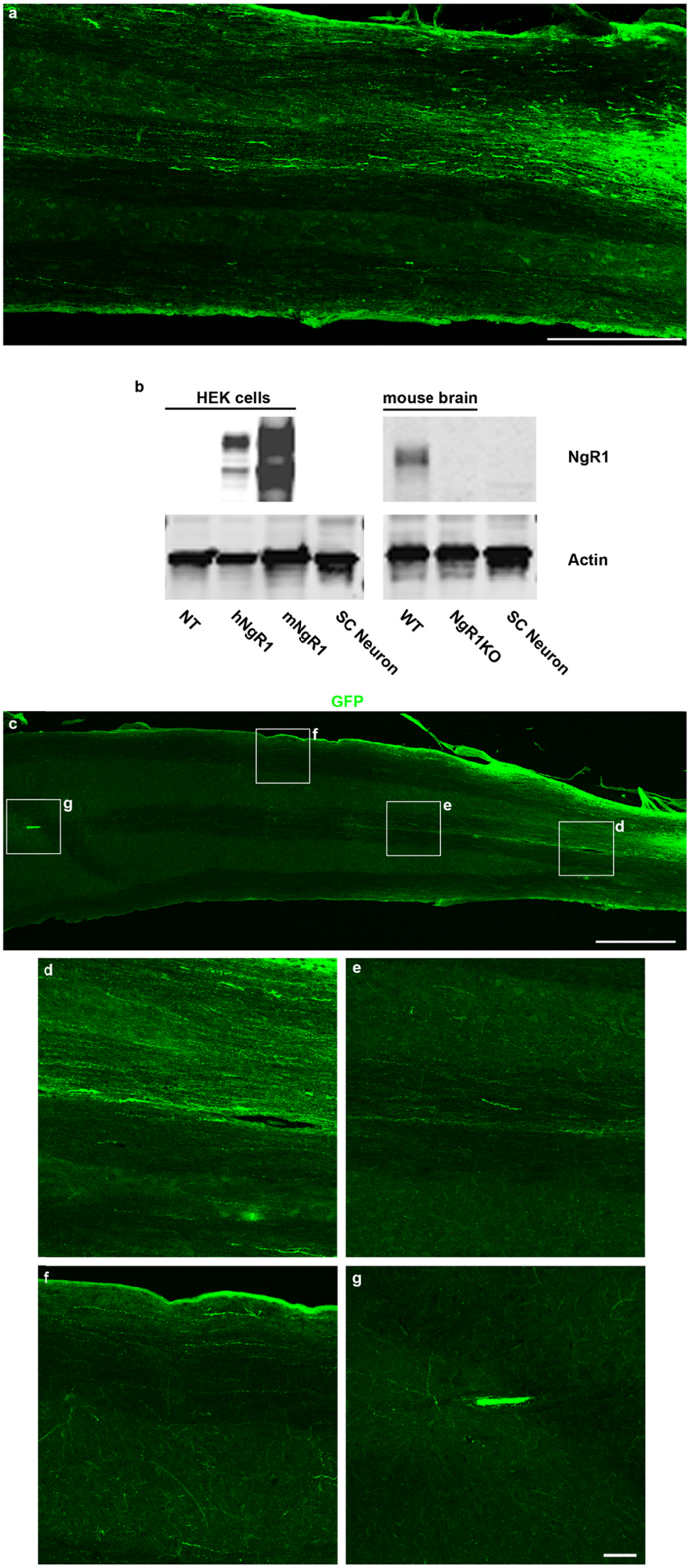
a
b
HEK cells
mouse brain
NgR1
Actin
NT
hNgR1
mNgR1
SC Neuron
WT
NgR1KO
SC Neuron
GFP
c
f
g
e
d
d
e
f
g

Supplementary Fig. 3. Axonal elongation from human SC-NES cell-derived neurons

(a) GFP immunostaining at graft-host interface showing extensive axonal elongation in the host SC. (b) Western blot performed on HEK cells and mouse brain lysates. HEK cells were harvested after transfection with the human (h) and murine (m) Nogo Receptor 1 (NgR1) constructs. The blot shows the lack of NgR1 expression in SC-NES cell-derived neurons (SC Neuron) as well as in non-transfected (NT) cells. Similarly, the blot on mouse wild type (WT) and NgR1 knock-out (KO) brains confirms the absence of NgR1 expression in human SC Neurons as well as in KO animals. (c) GFP immunostaining on a SC-NES cell recipient SC section showing long-distance axons originating from the main body of the graft in the lesion area (on the right). (d-g) High magnification images of insects in picture (c) showing GFP-labelled axons diving into the host SC at several distances from the main graft. The ectopic cluster of cells (g) appears clustered and lacking of long-distance projections. Scale bars: a, 500 μm; c, 1000 μm; d-g, 100 μm.

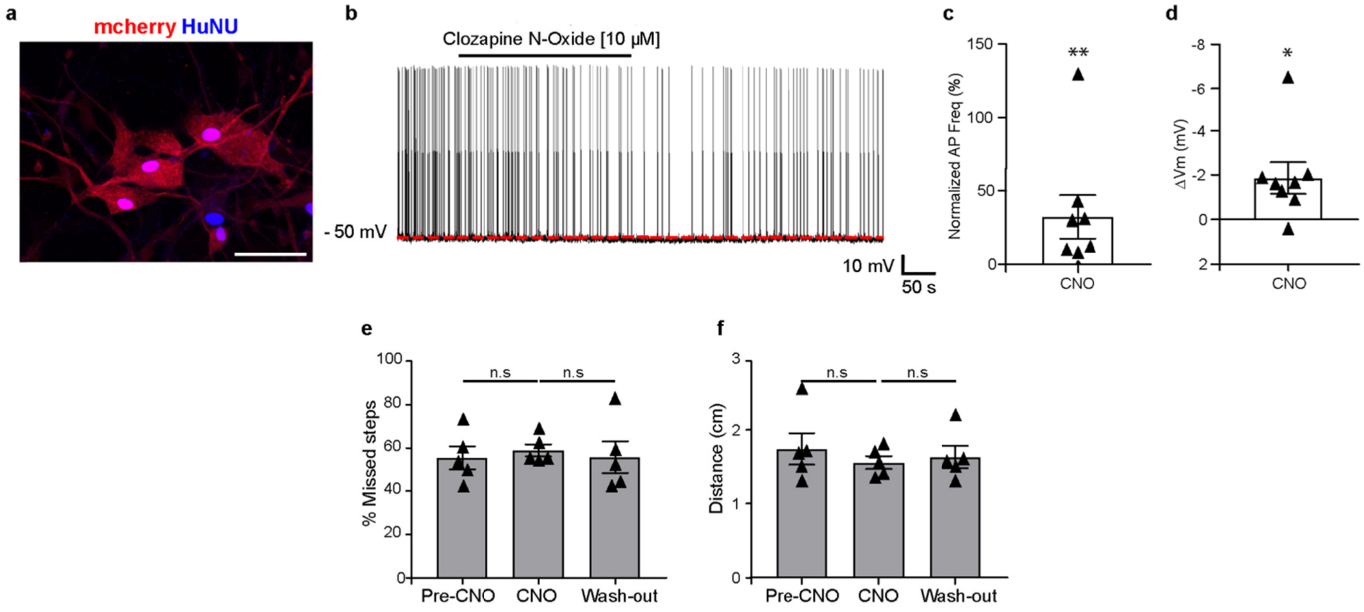

Supplementary Fig. 4. DREADD hM4Di receptor mediates neuronal silencing in human SC-NES cells

(a) mCherry and human nuclei (HuNu) immunostaining of SC-NES-derived neurons transduced with the hM4Di-mcherry lentiviral construct. (b) Perfusion with 10 μM CNO (continuous black line over the track) induces membrane hyperpolarization and reduction of spontaneous action potential frequency (n=8). Red dotted line indicates the membrane potential before drug perfusion. (c-d) Bar plots showing normalized action potential (AP) frequency variation (c) and membrane potential variation (d) induced by CNO. (e) Percentage of missed steps in the grid-walking test of control animals before, during and after CNO effect. (f) Quantification of stride length in control mice before, during and after CNO administration. Data represent means ± s.e.m. (* $P < 0.05$; ** $P < 0.01$; Student's t test). Scale bar a, 50 μm.

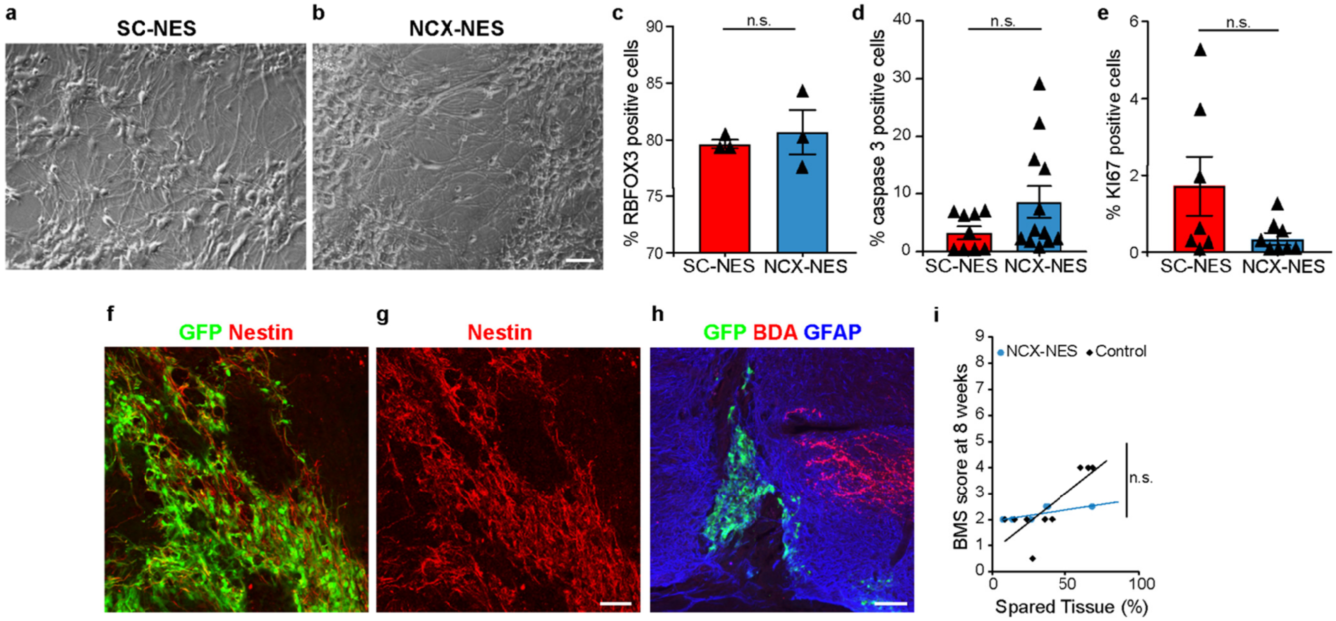

Supplementary Fig. 5. Human NCX-NES cell characterization in the injured SC

(a,b) Bright-field contrast images of SC-NES and NCX-NES cells after two months of *in vitro* differentiation. (c) Quantification of SC-NES and NCX-NES cell-derived RBFOX3-positive neurons *in vitro* showing no difference in the intrinsic neurogenic properties of the cells. (d) Quantification of activated caspase 3-positive cells in NCX- and SC-grafts shows no significant difference in the number of apoptotic cells. (e) Quantification of KI67-positive cells in NCX- and SC-grafts showing no significant difference in the number of proliferating cells. (f,g) Immunostaining of GFP-positive NCX-NES cells shows that the majority of the cells are immunoreactive for nestin. (h) Immunostaining of GFP-positive NCX-NES cells in the lesion core of the SC. GFAP staining indicates the glial scar. BDA stains the host CST and shows absence of axonal growth in the graft. Rostral is to the right. Caudal is to the left. (i) Correlation analysis of spared tissue percentage and BMS score at 8 weeks. Each dot represents an animal. The correlation shows no significant difference between NCX-NES cell recipient animals and the control group. Data are expressed as mean ± s.e.m. Statistics was performed using the Student’s t-test. Scale bars: a-b, 20 μm; f-g, 50 μm; h, 100 μm.

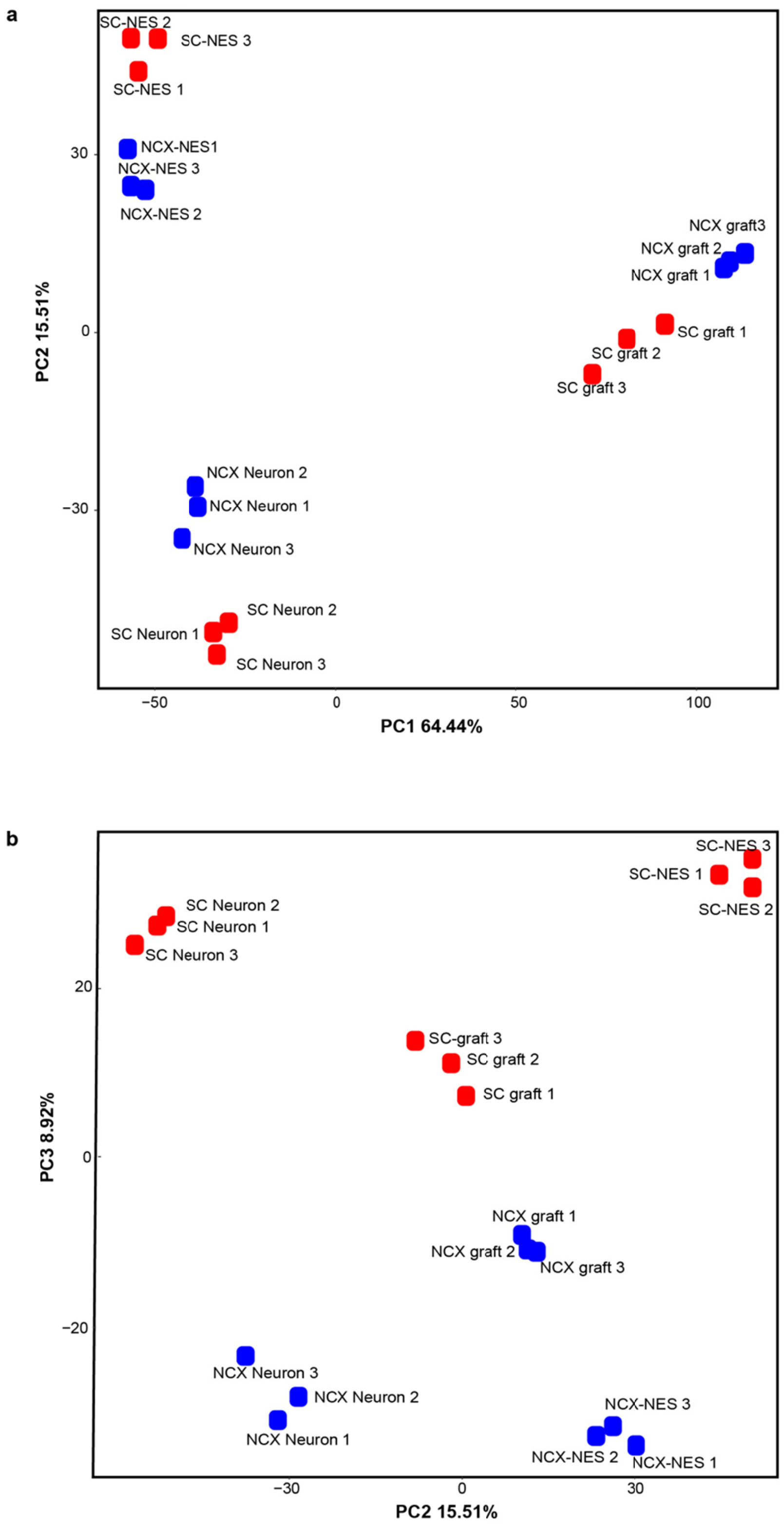

Supplementary Fig. 6. Principal component analysis of transcriptional profile of NES cells.

(a,b) Bidimensional plots of principal component analysis (PCA). Each symbol in red represents a SC sample including SC-NES (cells in proliferation), SC Neuron (*in vitro* differentiation) and SC graft (*in vivo* differentiation). Similarly, blue dots indicate NCX samples in the same conditions: NES, Neuron and graft. Each sample is in triplicate. PC1 segregates the *in vitro* from the *in vivo* samples while PC3 separates samples according to their anatomical origin.

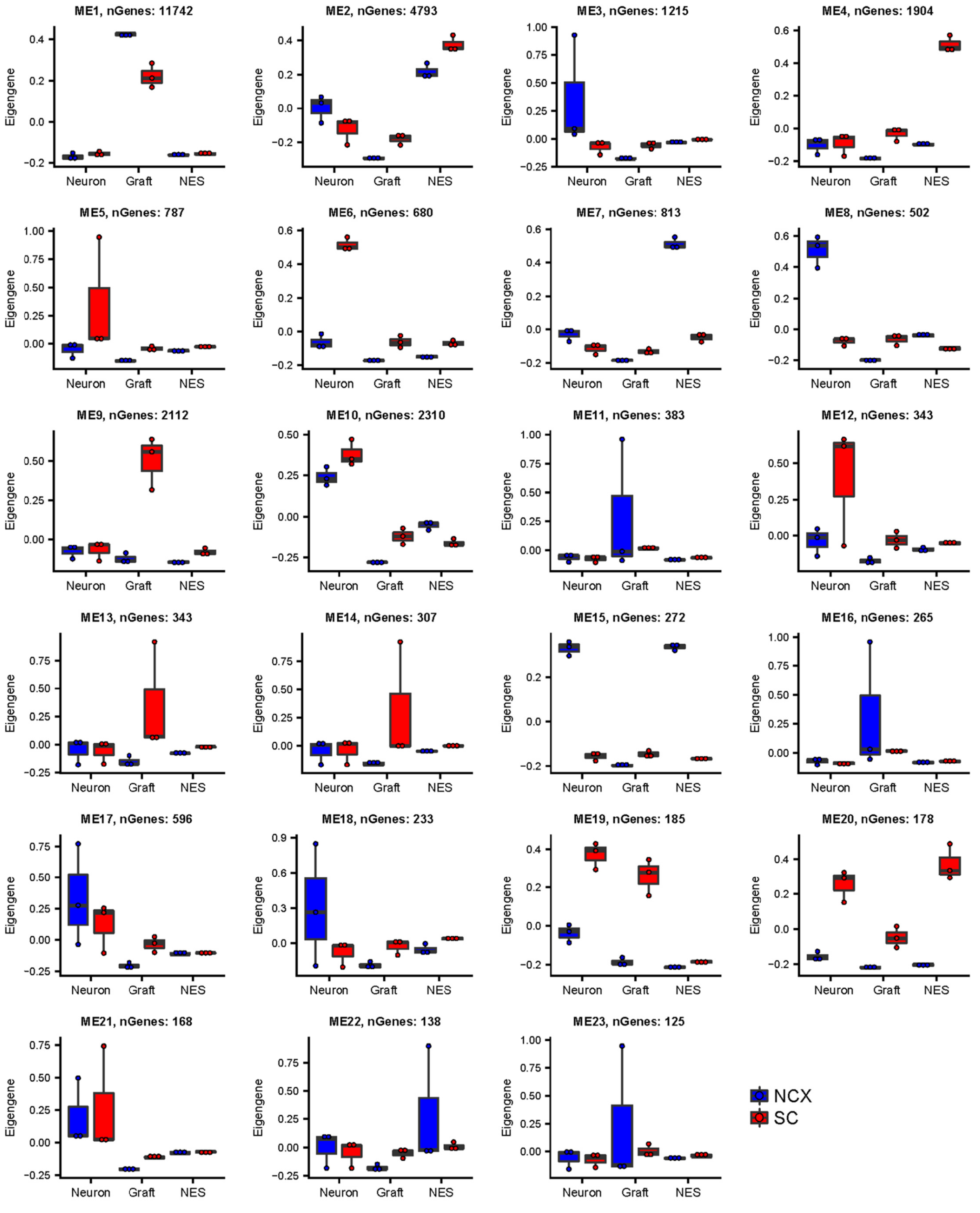

Supplementary Fig. 7. Module expression patterns in SC- and NCX-NES cells

Box plots depicting the expression patterns of 23 modules from NCX- and SC-NES cells in red and blue, respectively. Module eigengene (ME) was used to represent the expression pattern across the samples.

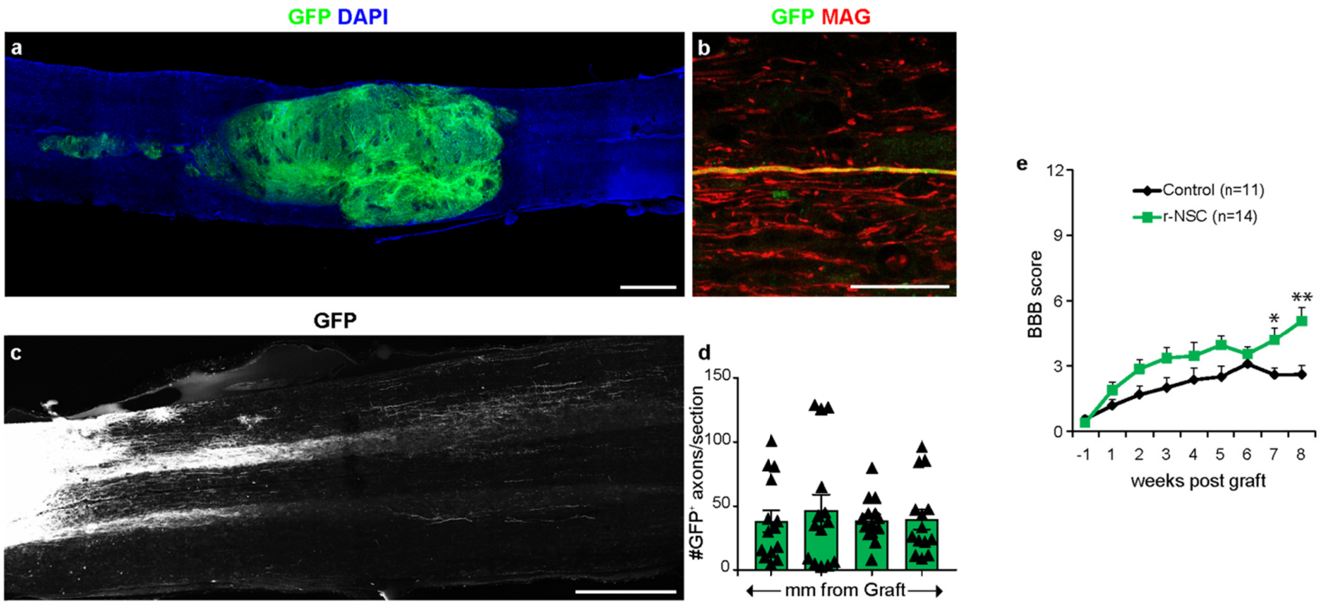

Supplementary Fig. 8. r-NSC graft charcterization in the contused SC

(a) GFP staining of r-NSCs in a rat contused SC showing cell surviving 2 months after transplantation. (b,c) r-NSCs integrate in the recipient tissue and extend a high number of axons which also appear to be myelinated by the host oligodendrocytes, as proved by the immunostaining for myelin associated glycoprotein (MAG). (d) Quantification of GFP-positive axons per section in the SC of recipient rats. (e) BBB score of recipient rats shows an improvement in the functional outcome of animals injected with cells compared to controls. The performance is significantly different from controls at 7 and 8 weeks after transplantation. Data are expressed as mean ± s.e.m (* $P < 0.05$; ** $P < 0.01$, *** $P < 0.001$; Repeated measures ANOVA was performed for the BBB test). Scale bars: a, c, 1000 μm; b, 50 μm.
