# Supplementary material for: Human neuroepithelial stem cell regional specificity enables spinal cord repair through a relay circuit
Source: Nat Commun. 2018 Aug 24;9:3419. doi: 10.1038/s41467-018-05844-8 (PMC6109094; doi:10.1038/s41467-018-05844-8)
Supplement: Supplementary file 3 — Description of Additional Supplementary Files [file 41467_2018_5844_MOESM3_ESM.pdf]

Description of Additional Supplementary Files

File Name: **Supplementary Data 1**
Description: Supplementary Data 1 provides detailed information on the Gene Modules identified by weighted gene co-expression correlation network analysis (WGCNA) and depicted in Supplementary Figure 7. For each Module, the statistically enriched Gene Ontology (GO) terms is listed, together with the individual genes represented for that Module and GO term.
